# Serum zinc associated with immunity and inflammatory markers in Covid-19

**DOI:** 10.1515/med-2022-0469

**Published:** 2022-04-07

**Authors:** Hassan Joulaei, Parisa Keshani, Zohre Foroozanfar, Daniel Zamanian, Amirhossein Hassani, Fateme Parvizi, Yasaman Khadem, Navid Omidifar, Mohammad Ali Davarpanah

**Affiliations:** Health Policy Research Center, Institute of Health, Shiraz University of Medical Sciences, Shiraz, Iran; HIV/AIDS Research Center, Institute of Health, Shiraz University of Medical Sciences, Shiraz, Iran; School of Medicine, Shiraz University of Medical Sciences, Shiraz, Iran; Clinical Education Research Center, and Department of Pathology, School of Medicine, Shiraz University of Medical Sciences, Shiraz, Iran

**Keywords:** serum zinc, interleukin-6, interferon-gamma, duration of hospitalization, mortality rate, Covid-19

## Abstract

This study aimed to assess the association between serum zinc level with some inflammatory and immunity factors and the duration of hospitalization and mortality rate in patients diagnosed with Covid-19. In this cross-sectional study, blood samples were taken from polymerase chain reaction (PCR) positive patients. New patients diagnosed with Covid-19, admitted to different public hospital wards, were considered eligible for entering the study. The study was done on 179 hospitalized patients diagnosed with Covid-19. Fourteen patients died during the hospitalization and the in-hospital mortality rate was 7.8%, with 9.1% (13 patients) of patients with serum zinc level less than 70 mcg/dL and 3.4% (1 patient) of patients with zinc levels more than 70 mcg/dL. Higher levels of zinc were significantly associated with a higher and lower level of interferon-gamma (IFN-γ) (*p*-value = 0.035) and interleukin (IL)-6 (*p*-value = 0.004), respectively. The level of serum zinc did not have a significant association with mortality even after adjusting for confounding factors. The relationship between zinc level and the duration of hospitalization was also not significant. In conclusion, serum zinc level had an association with IL-6 and IFN-γ level, but it did not have any significant association with hospital duration or mortality.

## 1 Introduction

Severe Acute Respiratory Syndrome Coronavirus 2 (SARS-CoV-2), the cause of coronavirus disease 2019 (Covid-19), was first reported in China in late 2019 from a zoonotic source [1]. The full spectrum of Covid-19 ranges from mild, self-limiting respiratory tract illness to severe progressive pneumonia, multi-organ failure, and death [2]. After an incubation period, the acute viral phase in patients with symptomatic Covid-19 usually manifests as influenza-like symptoms. In some persons, the illness progresses to hypoxemic respiratory failure [3]. Evidence suggests that the pathophysiological basis of this profound decline is a severe inflammatory response resembling cytokine release syndrome [4,5]. In this phase, patients have markedly abnormal inflammatory markers, including elevated serum interleukin (IL)-6, ferritin, and C-reactive protein (CRP) levels [6,7,8]. Zinc (Zn) is an essential micronutrient that is involved in the regulation of innate and adaptive immune responses [9]. Zn is involved in a variety of biological processes due to its function as a cofactor, signaling molecule, and structural element. The most critical role of Zn is demonstrated in the immune system. Zn regulates the proliferation, differentiation, maturation, and functioning of leukocytes and lymphocytes [9]. Zn also plays a signaling role involved in the modulation of inflammatory responses [9]. Having said the role of Zn in inflammatory responses, there must be an understanding of cytokines. Cytokines are one term for a group of protein cell regulators, variously called lymphokines, monokines, IL, and interferons (IFN), which are produced by a wide variety of cells in the body. They play an important role in many physiological responses, involved in the pathophysiology of a range of diseases, and have a therapeutic potential [10]. Evidence has shown the role of different types of cytokines in Covid-cytokine storm syndrome (COVID-CSS). In CSS, many inflammatory cytokines such as IL-1, IL-10, and tumor necrosis factor (TNF)-α are elevated, approximately 2–100 folds above normative values, whereas IL-6 demonstrates much larger increases, in some cases more than 1000 folds above normal [11]. Other cytokines such as interferon-gamma (IFN-γ) is capable of orchestrating numerous protective functions to heighten immune responses in infections and cancers [12]. Zn deficiency results in altered numbers and dysfunction of all immune cells, subjects with suboptimal Zn state have an increased risk of infectious diseases and autoimmune disorders [11,13,14,15]. Zn deficiency is responsible for 16% of all deep respiratory infections worldwide [16]. During chronic Zn deficiency, the production of pro-inflammatory cytokines increases, influencing the outcome of a large number of inflammatory, metabolic, neurodegenerative, and immune diseases [17]. Zn is essential to preserve natural tissue barriers such as the respiratory epithelium, preventing pathogen entry, for a balanced function of the immune and redox system. There are several data surrounding the association between Zn status and viral respiratory tract infections, but there is still more to know regarding its association with Covid-19. It has also been suggested that most Covid-19 symptoms are associated with altered Zn homeostasis and Zn might even prevent or attenuate those symptoms and thus should be regarded as a promising cost-effective, globally available therapeutic approach for Covid-19 patients [18]. Given all the data surrounding the role of minerals, especially Zn in the regulation of immune responses, especially against respiratory tract infections and the unprecedented situation we are living in, with a global pandemic affecting many lives and causing many more hospitalizations, without much effective prophylaxis or treatment against Covid-19, studying the concentration of Zn in individuals affected with this disease and its association with some immunological and inflammatory factors might help us develop more accurate treatments. Prophylaxis against this disease can help us get into a deeper understanding of the role of minerals, especially Zn in Covid-19 and other similar respiratory infections. This study aimed to assess the association between serum Zn levels and some inflammatory and immunity factors and also to evaluate the association between serum Zn level and the duration of hospitalization and mortality rate in patients diagnosed with Covid-19.

## 2 Methods

### 2.1 Participants

In this cross-sectional study blood samples were taken from 179 polymerase chain reaction (PCR)-positive patients. New patients diagnosed with Covid-19, admitted to different public hospital wards, were considered eligible for entering the study. Demographic and lab data were also collected at the time of admission. The duration of hospitalization and the mortality rate of these patients were also monitored. The patients were required to fill and sign in a consent form that explained the goal of the study. The information gathered from the patients included: name and address, telephone number, hospitalization document code, date and duration of hospital stay, date of death, sex and age, height and weight, educational status, job status, past medical history such as (hypertension, dyslipidemia, diabetes, underlying heart or vascular disease, chronic lung and airway diseases such as asthma, cancer, etc.), drug history (including the history of vitamin or mineral supplement intake), alcohol and smoking history, and hospital prescriptions (antibiotics, corticosteroids, respiratory drugs, etc.) were gathered by interviewing and using hospital documents. Biochemical parameters such as oxygen saturation (SpO_2_), complete blood count (CBC), erythrocyte sedimentation rate (ESR), CRP, liver and kidney function tests, ferritin, and lactate dehydrogenase (LDH) were also gathered from hospital documents at the time of admission, and serum Zn, IL-6, and IFN-γ level were measured.

### 2.2 Measurements

The samples were taken from patients hospitalized at the internal ward and emergency room (ER). 3cc of blood was drawn from each individual and was put in a clot activator tube. The tubes were then put in a centrifuge with a rotational speed of 4,000 rpm for 5 min. The residual plasma for each patient was transferred to a micro-tube and stored at −70°C until the end of the study.

#### 2.2.1 Serum Zn

Serum Zn was measured using AA500 Atomic Absorption Spectrometer. Given the lack of adequate data on the Zn deficiency status among Iranian individuals, the reference amount of Zn mentioned in the textbooks and articles which are between 70 and 120 mcg/dL were set as the normal range for individuals [19]. Those with serum Zn levels less than 70 mcg/dL were considered as Zn deficient.

#### 2.2.2 Serum IL-6

The IMMULITE 2000 Systems Analyzer was used for the quantitative measurement of IL-6 in serum samples. IMMULITE 2000 IL-6 is a solid-phase, enzyme-labeled, chemiluminescent sequential immunometric assay. The absolute range was reported from nondetectable to 5.9 pg/mL for the procedure and analytical sensitivity was 2 pg/mL.

#### 2.2.3 Serum IFN-γ

ZellBio ELIZA kit (Germany) was used for a quantitative assay of human IFN-γ on the basis of the Biotin double-antibody sandwich technology. Sensitivity was 1.5 ng/mL and the assay range was from 12.5 to 400 ng/mL.

### 2.3 Statistical analyses

Quantitative variables were reported as mean value ± standard deviation and qualitative variables were described in terms of number and percentage. Chi-square and *t*-tests were used to compare demographic and clinical characteristics in the Covid-19 Patients (alive or dead).

In order to determine the contribution of the Zn as a predictor of inflammatory factors (IFN-γ, IL-6, ESR, CRP, white blood cells (WBC), neutrophils, lymphocytes, SpO_2_, and LDH) in the Covid-19 patients, linear regression analysis was used. Logistic regression was used to determine the association between Zn and confounding variables with death outcome. Linear regression was also used to determine the relationship between Zn and confounding variables with the duration of hospitalization results.

Data were analyzed by SPSS software version 22 and GraphPad Prism software version 8. Also, *p*-value <0.05 was considered as the statistically significant level.


**Ethical standards disclosure:** This study was conducted according to the guidelines laid down in the Declaration of Helsinki and all procedures involving research study participants were approved by the Shiraz University of Medical Sciences (SUMS) ethics board committee, reference number: IR.SUMS.REC.1399.836. Written informed consent was obtained from all participants and questionnaires were anonymous and encoded.

## 3 Results

In this study, 179 hospitalized patients diagnosed with Covid-19 participated. The mean age of the participants was 55.27 ± 16.23 in the total sample, with 54.30 ± 15.78 in survivor patients, and 66.79 ± 17.51 in non-survivor patients. This difference was statistically significant (*p* = 0.001). There were 108 (60.3%) male patients in the study. Among all the patients, 140 (81.9%) patients had Zn levels under 70 mcg/dL in which 83 (82.2%) of the male and 57 (81.4%) of the female population were Zn insufficient or deficient. Hypertension was the most common underlying disease in the participants (39.1% of patients reported a history of hypertension) and dyspnea was the most common presenting symptom in the patients diagnosed with Covid-19 (dyspnea has been reported in 78.2% of patients). About one-quarter of the patients had a history of vitamin use (50, 27.9%). Other demographic and clinical information of patients are reported in Table 1.

**Table 1:** Characteristics of patients with Covid-19 by survival status

Characteristics	Patients (*n* = 179)	Survived (*n* = 165)	Non-survived (*n* = 14)	*p* Value
Age mean value ± SD	55.27 ± 16.23	54.30 ± 15.78	66.79 ± 17.51	0.001*
BMI (kg/m^2^) mean value ± SD	27.54 ± 5.32	27.63 ± 5.46	26.37 ± 2.63	0.444
Sex *N* (%)				
Male	108 (60.3)	97 (58.8)	11 (78.6)	0.159
Female	71 (39.7)	68 (41.2)	3 (21.4)	
Education *N* (%)				
Illiterate	34 (19.0)	33 (19.4)	2 (14.3)	0.544
Primary school	38 (21.2)	35 (21.2)	3 (21.4)	
Guidance school	13 (7.3)	10 (6.1)	3 (21.4)	
High school and diploma	30 (16.8)	30 (18.2)	—	
Undergraduate	27 (15.1)	26 (15.8)	1 (7.1)	
Post graduate	13 (7.3)	12 (7.3)	1 (7.1)	
Occupation *N* (%)				
Unemployed	4 (2.2)	4 (2.4)	—	0.781
House wife	59 (33.0)	56 (33.9)	3 (21.4)	
Worker	8 (4.5)	7 (4.2)	1 (7.1)	
Employed	28 (15.6)	26 (15.8)	2 (14.3)	
Self-employed	46 (25.7)	43 (26.1)	3 (21.4)	
Retired	19 (10.6)	16 (9.7)	3 (21.4)	
Student	1 (0.6)	1 (0.6)	—	
Current smoking *N* (%)	39 (22.2)	38 (23.0)	1 (7.1)	0.190
Alcohol consumer *N* (%)	15 (8.5)	15 (9.1)	—	0.273
Place of residence *N* (%)				
Urban	147 (82.1)	135 (82.3)	12 (85.7)	0.691
Rural	30 (16.8)	28 (17.1)	2 (14.3)	
Vitamin supplement consumption *N* (%)	50 (27.9)	47 (28.8)	47 (28.8)	0.557
Vitamin C intake *N* (%)	16 (8.9)	15 (9.1)	15 (9.1)	0.807
Vitamin D intake *N* (%)	27 (15.1)	25 (15.2)	25 (15.2)	0.931
Zinc <70 mg/dL *N* (%)	142 (79.3)	130 (78.8)	12 (85.7)	0.316
History of underlying disease *N* (%)				
Diabetes	52 (29.2)	46 (28.0)	6 (42.9)	0.249
Hypertension	70 (39.1)	62 (37.6)	8 (57.1)	0.158
Cardiovascular disease	56 (31.3)	49 (29.7)	7 (50.0)	0.125
Asthma and respiratory disease	22 (12.5)	20 (12.3)	2 (14.3)	0.827
Cancer	7 (3.9)	6 (3.6)	1 (7.1)	0.618
Autoimmune disease	16 (8.9)	9 (5.5)	—	0.472
HIV	2 (1.1)	2 (1.2)	—	0.849
Duration of hospitalization median (IQ)	3 (2–5)	3 (2–5)	2.50 (1.75–6.25)	0.838
Laboratory examinations mean value ± SD				
Zinc (mcg/dL) (*n* = 171)	58.65 ± 13.18	58.98 ± 13.38	54.61 ± 9.96	0.251
IFN-γ (ng/mL) (*n* = 171)	19.04 ± 19.88	19.12 ± 20.4	18.11 ± 18.51	0.860
IL-6 (pg/mL) (*n* = 170)	33.68 ± 40.21	32.80 ± 40.77	44.26 ± 31.99	0.330
LDH (IU/L) (*n* = 153)	728.92 ± 397.74	720.15 ± 407.08	823.31 ± 271.67	0.376
RBC (×10^6^/µL) (*n* = 179)	4.73 ± 0.69	4.69 ± 0.69	5.14 ± 0.64	0.024*
HB (g/dL) (*n* = 179)	13.15 ± 2.06	13.01 ± 2.01	14.77 ± 2.13	0.003*
HCT (%) (*n* = 179)	40.73 ± 5.6	40.34 ± 5.48	45.32 ± 5.05	0.002*
WBC (×1,000/µL) (*n* = 179)	6.85 ± 3.39	6.77 ± 3.47	7.76 ± 2.07	0.302
PLT (×1,000/µL) (*n* = 179)	216.24 ± 118.72	218.21 ± 121.43	193.07 ± 79.73	0.437
Neutrophils (%) (*n* = 176)	76.23 ± 57.17	75.87 ± 59.54	80.37 ± 8.08	0.781
Lymphocytes (%) (*n* = 177)	20.74 ± 10.48	21.38 ± 10.47	13.22 ± 7.29	0.008*
ESR (mm/h) (*n* = 128)	49.26 ± 26.53	49.04 ± 26.20	51.41 ± 30.66	0.767
CRP (mg/L) (*n* = 164)	36.65 ± 32.97	35.64 ± 32.66	48.41 ± 35.57	0.186
SpO_2_ (%) (*n* = 176)	86.48 ± 11.08	87.25 ± 10.19	77.64 ± 15.95	0.005*
FBS (mg/dL) (*n* = 91)	146.12 ± 68.81	143.77 ± 67.19	161.58 ± 80.17	0.406
BUN (mg/dL) (*n* = 179)	19.53 ± 10.96	19.29 ± 11.23	22.43 ± 6.84	0.309
Creatinine (mg/dL) (*n* = 179)	1.26 ± 0.67	1.26 ± 0.69	1.31 ± 0.26	0.739
Uric acid (mg/dL) (*n* = 80)	5.49 ± 2.55	5.69 ± 2.57	4.24 ± 2.10	0.081
S.G.O.T (IU/L) (*n* = 164)	53.63 ± 54.74	53.43 ± 59.0	55.85 ± 21.04	0.874
S.G.P.T (IU/L) (*n* = 164)	43.35 ± 36.40	44.11 ± 37.76	35.21 ± 13.73	0.388
PTT (s) (*n* = 166)	37.94 ± 15.01	38.53 ± 15.50	31.57 ± 4.84	0.035*
INR (*n* = 13)	1.22 ± 0.39	1.15 ± 0.15	1.14 ± 0.15	0.360
D-dimer (ng/mL) (*n* = 79)	1,679.02 ± 1,850.33	1,699.43 ± 1,924.89	—	0.770
Ferritin (ng/mL) (*n* = 36)	742.80 ± 694.65	731.74 ± 722.38	—	0.750
Treatments				
Covid-19 drug	153 (85.5)	139 (84.2)	14 (100.0)	0.101
Other antibiotics	135 (85.5)	142 (86.1)	11 (78.6)	0.450
Corticosteroids	122 (68.2)	111 (67.3)	11 (78.6)	0.389
Respiratory drugs	100 (55.9)	90 (54.5)	10 (71.4)	0.231

*p < 0.05. # Covid-19 drugs include: favipiravir, efavirence, zidovudine, lamivudine, lopinavir, ritonavir, atazanavir, kaletra, remdesivir, linezolid, IFN-beta, ivermectin, HCQ. Other antibiotics include: imipenem, meropenem, tavanex, ampibactam, vancomycin, azithromycin, clexane, ceftriaxone, clindamycin, metronidazole, coamoxiclav, ampisulbactam, ampicillin, sulbactam. Corticosteroids include: dexamethasone, prednisolone, methylprednisolone, hydrocortisone, triamcinolone, pulmicort neb. Respiratory drugs include: salbutamol, atrovent, seroflo, guaifenesin, carnitine, bromhexine, dextromethorphan, theophylline, pirfenidone, tiova. BMI, body mass index; BUN, blood urea nitrogen; CRP, C-reactive protein; ESR, erythrocyte sedimentation rate; FBS, fasting blood sugar; HB, hemoglobin; HCT, hematocrit; HIV, human immunodeficiency virus, IFN-γ, interferon-gamma; IL-6, interleukin-6; INR, international normalized ratio; IQ, interquartile; IU, international unit; LDH, lactate dehydrogenase; PLT, platelets; PTT, partial thromboplastin time; RBC, red blood cells; S.G.O.T, serum glutamic-oxaloacetic transaminase; S.G.P.T, serum glutamic-pyruvic transaminase; SpO_2_, oxygen saturation; WBC, white blood cells.

Fourteen patients died during the hospitalization and the in-hospital mortality rate was calculated as 7.8%, and 9.1% (13 patients) of patients had serum Zn level less than 70 mcg/dL and 3.4% (1 patient) of patients had serum Zn level more than 70 mcg/dL; however, this difference was not statistically significant (*p* = 0.331). The median duration of hospitalization was 3 days (2–5 days) which happened to be the same for patients with Zn levels lower or higher than 70 mcg/dL. According to the results, there was a significant association between serum Zn and IFN-γ (*p*-value = 0.035), a higher level of Zn was significantly associated with a higher level of IFN-γ. A significant association was also found between Zn level and IL-6 (*p*-value = 0.004). Higher levels of serum Zn were significantly associated with a lower level of IL-6. There was no significant association between Zn level and other inflammatory factors (Table 2). Zn did not have a significant association with death. Even after adjustment for confounding factors such as age as well as history of underlying disease, there still happened to be no significant association between Zn and mortality rate. The relationship between serum Zn level and the duration of hospitalization was not significant either. Even after adjusting for confounding factors (age and history of underlying disease), there was still no significant association between serum Zn level and the duration of hospitalization (Table 3).

**Table 2:** Association between zinc and inflammatory factors

Independent variable and outcome	*B*	SE	*p*-value
Zinc and IFN-γ	0.25	0.12	0.035*
Zinc and IL-6	−0.70	0.24	0.004*
Zinc and ESR	1.53	0.79	0.084
Zinc and CRP	1.61	0.98	0.131
Zinc and WBC	−0.04	0.06	0.501
Zinc and neutrophils	0.12	0.25	0.628
Zinc and lymphocytes	−0.02	0.23	0.934
Zinc and SpO_2_	0.001	0.48	0.999
Zinc and LDH	0.84	0.50	0.745

*Significant at 0.05 level; zinc is considered as an independent variable. *B*, linear regression coefficient; CRP, C-reactive protein; ESR, erythrocyte sedimentation rate; IFN-γ, interferon-gamma; IL-6, interleukin-6; LDH, lactate dehydrogenase; SE, standard error; SpO_2_, oxygen saturation; WBC, white blood cells.

**Table 3:** Factors associated with duration of hospitalization and survival

	Duration of hospitalization			Death		
	*B*	SE	*p* value	OR	95% CI	*p* value
Model 1 (simple model)						
Zinc	−0.01	0.01	0.541	0.97	0.93–1.02	0.251
Model 2 (multiple model)						
Zinc	−0.01	0.01	0.845	0.97	0.93–1.03	0.406
Age	0.02	0.01	0.041*	1.06	1.01–1.10	0.010*
History of underlying disease						
No	Ref.	—	—	Ref.	—	—
Yes	0.61	0.41	0.132	1.71	0.19–14.86	0.626

*Significant at 0.05 level; CI: confidence interval. *B*, regression coefficient; OR, odds ratio; SE, standard error.

**Figure 1:**
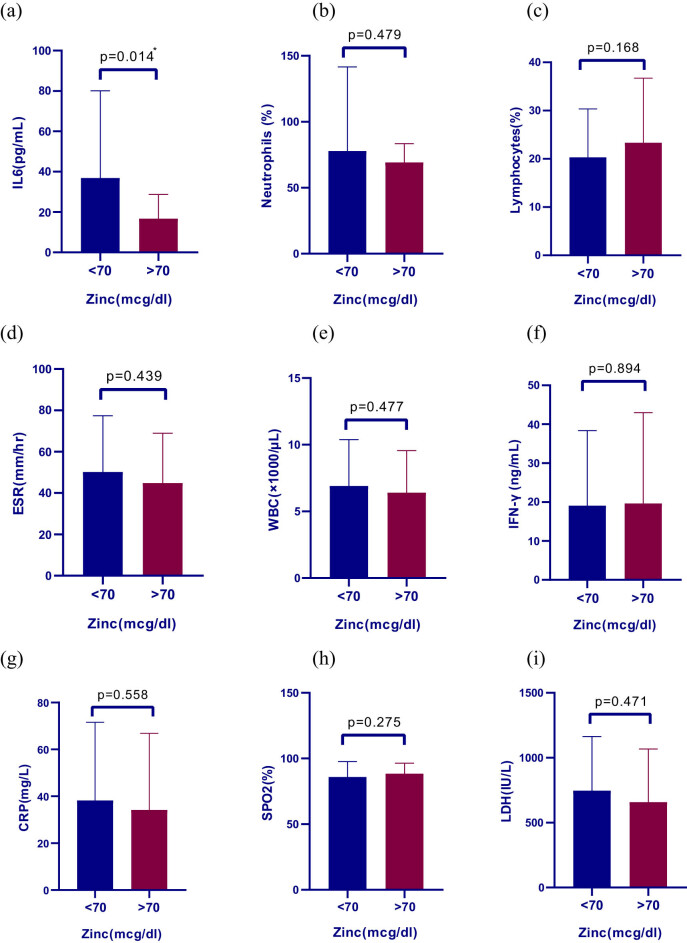
Comparison of inflammatory factors in two subgroups of serum zinc (less and more than 70 mcg/dL). (a) Serum zinc and IL-6 (pg/mL. (b) Serum zinc and neutrophils (%). (c) Serum zinc and lymphocytes (%). (d) Serum zinc and ESR (mm/h). (e) Serum zinc and WBC (×1,000/µL). (f) Serum zinc and IFN-γ (ng/mL). (g) Serum zinc and CRP (mg/L). (h) Serum zinc and SpO_2_ (%). (i) Serum zinc and LDH (IU/L).

**Figure 2:**
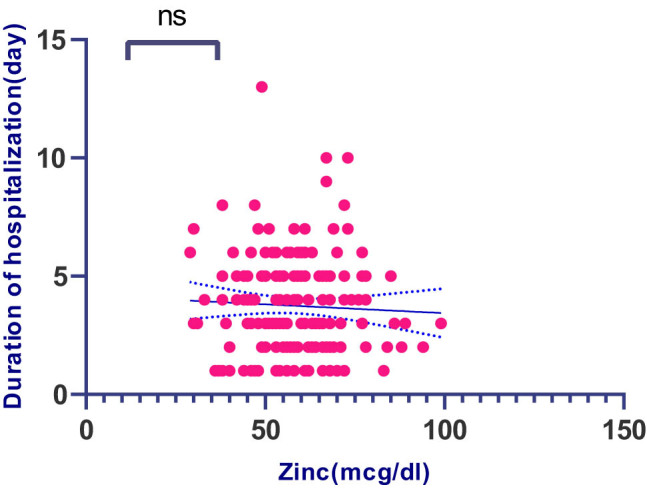
Correlation between serum zinc level and duration of hospitalization.

The patients were divided into two groups based on their Zn status (less and more than 70 mcg/dL). The inflammatory factors in the two subgroups are shown in Figure 1. According to the results, there was no significant difference in inflammatory factors between the two subgroups based on serum Zn (less and more than 70 mcg/dL) except for IL-6 (*p*-value = 0.014). The amount of IL-6 in patients with serum Zn levels less than 70 mcg/dL was significantly higher than patients with Zn levels more than 70 mcg/dL. Correlation between serum Zn level and duration of hospitalization is shown in Figure 2.

## 4 Discussion

Given the novelty of the SARS-CoV-2 disease, not many studies have been reported regarding the measurement of serum Zn level and comparing it with inflammatory factors such as IL-6 or IFN-γ in patients diagnosed with Covid-19, but the role of Zn in inflammation and immunity has been presented in previous studies. Proliferation, differentiation, maturation, and functioning of leukocytes including lymphocytes are all regulated by Zn [7]. Chronic inflammation is characterized by an increased level of inflammatory cytokine production and Zn deficiency could cause altered production of cytokines which could lead to inflammation [20]. Lower circulating Zn level correlates with increased IL-6, IL-8, and TNF-α, in addition, cytokine signaling pathways are influenced by Zn status [21]. Cytokines are produced by different types of cells, primarily by T-lymphocytes and macrophages and the influence of Zn on these cells may explain the observed effect Zn has on cytokine production [22,23]. Zn deficiency is associated with downregulation of IFN-γ, resulting in severe impairment of cell-mediated immunity [15]. As mentioned, regarding the association between Zn and inflammatory factors in our study, 2 inflammatory factors had a significant association (*p*-value <0.05) with increased Zn level, higher level of Zn were significantly associated with a lower level of IL-6 (*p*-value = 0.004) and a higher level of IFN-γ (*p*-value = 0.035) which are in accordance with the previous studies. Other inflammatory factors that we studied were: ESR, CRP, WBC, neutrophils, lymphocytes, SpO_2_, and LDH. The association of all these other factors with increased serum Zn level was not statistically significant (*p* > 0.05). We also divided the patients into two groups regarding their Zn status and set the Zn level of 70 mcg/dL [24] as the cutoff value. In this division, the only inflammatory factor which was different between Zn sufficient and deficient groups was IL-6 (*p*-value = 0.014). Other factors that were measured and compared in these two groups were: WBC, lymphocytes, ESR, CRP, SpO_2_, and IFN-γ. Patients with Zn sufficient status (Zn > 70 mcg/dL) had a higher SpO_2_, lymphocytes, and IFN-γ but lower CRP and lower ESR compared to the Zn-deficient group. However, none of these differences were statistically significant. Serum Zn level did not have a statistically significant effect on mortality and hospitalization duration (*p* = 0.251). The only factor that had an effect on hospitalization and mortality was age with a *p*-value of 0.010. Thomas et al. conducted a randomized clinical trial study published in JAMA which recently concluded that the initiation of high-dose Zn gluconate, ascorbic acid, or a combination of the two supplements did not significantly decrease the duration of the symptoms or mortality compared with standard of care. This study also indicated that there was no significant difference in any secondary outcome such as number of days to reach no presence of fever, cough, shortness of breath, or fatigue in patients who received high dose Zn and ascorbic acid [25]. Based on our findings and a previous article, it seems that Zn levels do not have an effect on mortality and duration of hospitalization. The percentage of patients who died in this study was 7.8% with 9.1% being the mortality rate of patients with serum Zn levels less than 70 mcg/dL and 3.4% being the mortality rate of the patients with serum Zn levels higher than 70 mcg/dL. Although the mortality rate was higher in patients with serum Zn level less than 70 mcg/dL, the results were not statistically significant and the reason could be because of the disproportionate number of patients with serum Zn level less than 70 mcg/dL compared to those with serum Zn level higher than 70 mcg/dL. A study conducted by Nasrollahzadeh and colleagues in 2020 showed that the mortality percentage in their study was 21.9% but the study was done on Covid-19 patients who had an underlying disease [26] and it can explain the higher mortality rate reported in their study compared to this one. Our study has shown that there is an association between serum inflammatory markers and Zn level especially cytokine level such as IL-6 in patients with Covid-19. This association has been seen in other studies as mentioned above. Whether serum Zn level can have a subsequent effect on conditions such as cytokine storm in patients with Covid-19 warrants for further investigation and more study needs to be done on the relationship between serum Zn level in patients diagnosed with Covid-19 and other factors.

In this study, we also assessed several other laboratory data in the patients, such as WBC, red blood cells (RBC), hematocrit (HCT), platelets (PLT), neutrophil, lymphocyte, and SpO_2_. Among the factors mentioned above, four factors were statistically significant between the patients who lived and those who died as an endpoint for the study; RBC and HCT were higher in the patients who died and lymphocytes and SpO_2_ were higher in the patients who lived. Most Covid-19 fatalities experienced greater lymphopenia. Decreased lymphocytes accompanied by mild thrombocytopenia were among the most common abnormal findings attracting attention in the CBC of Covid-19 patients [27]. The lymphocyte count is an important parameter to directly discriminate between Covid-19 patients with and without the severe disease [22]. Other studies have also shown a higher level of neutrophils in patients with severe Covid-19 disease [27]. Our study demonstrated a statistically significant association between lymphocyte counts and mortality, with these counts being lower in patients who passed away, which is in line with the mentioned previous findings, but with regard to the platelet counts and neutrophils, we found no statistically significant association. With regards to the SpO_2_ level, patients who lived had higher SpO_2_ levels. This association was also demonstrated in another study conducted by Jiang Xie and coworkers in 2020. In this cohort of patients with Covid-19, hypoxemia was independently associated with in-hospital mortality [23]. Another study conducted by Celine Renoux and colleagues showed that RBC and HCT levels were higher in Covid patients compared to non-Covid sepsis but the results were not statistically significant. But the study showed a statistically significant association between RBC rheological properties in Covid-19 infected patients. A statistically significant association was found between RBC and HCT levels in this study [28]. We should also note that a correlation between serum Zn level and Covid-19 outcome has been demonstrated in a recent study by Vogel-González et al. There were 249 participants in this study and serum Zn levels lower than 50 µg/dL at admission correlated with worse clinical presentation, longer time to recovery, and higher mortality. This study also demonstrated a higher level of inflammatory markers, such as IL-6 and CRP, in patients with low serum Zn levels [29]. Relative to hospitalization duration and mortality rates, our study has shown otherwise, with no association between serum Zn level and these two factors in patients diagnosed with Covid-19. There were some limitations in the study. The number of patients who were considered Zn deficient was disproportionate compared to those regarded as Zn sufficient, thus this could have probably been the limitation of the study and the reasons for not finding statistically significant associations. The study also had its strengths, the hospital where our research was conducted is one of the main hospitals for admitting patients diagnosed with Covid-19 in a city with a population of roughly 2 million. The criteria for admission for all the patients were the same and the same guidelines were followed.

## 5 Conclusion

In summary, serum Zn level had an association with IL-6 and IFN-γ levels but no significant association between serum Zn level and hospital duration or mortality was found. Although higher mortality rates were reported among patients considered to have inadequate serum Zn levels, this result was not considered to be statistically significant. We believe that further studies still have to be conducted in order to bring us more solid evidence with regards to the association between serum Zn level and hospitalization duration and mortality. In measuring other factors in patients infected with Covid-19, our study demonstrated a significant association between RBC, HCT, lymphocytes, and SpO_2_ in the patients who lived and those who died. Although the association between Zn and inflammatory markers and its outcome on non-Covid sepsis and other infections has been demonstrated in numerous studies, the effect of Zn on these factors and other factors in patients diagnosed with Covid-19 warrants for further investigation.
